# Molecular Mechanisms of Antitumor Activity of PAMAM Dendrimer Conjugates with Anticancer Drugs and a Monoclonal Antibody

**DOI:** 10.3390/polym11091422

**Published:** 2019-08-29

**Authors:** Monika Marcinkowska, Maciej Stanczyk, Anna Janaszewska, Arkadiusz Gajek, Malgorzata Ksiezak, Paula Dzialak, Barbara Klajnert-Maculewicz

**Affiliations:** 1 Department of General Biophysics, Faculty of Biology and Environmental Protection, University of Lodz, Pomorska 141/143, 90-236 Lodz, Poland; 2 Department of Surgical Oncology, Cancer Center, Copernicus Memorial Hospital, 93-513 Lodz, Poland; 3 Department of Medical Biophysics, Faculty of Biology and Environmental Protection, University of Lodz, Pomorska 141/143, 90-236 Lodz, Poland; 4 Leibniz-Institut für Polymerforschung Dresden e.V., Hohe Strasse 6, 01069 Dresden, Germany

**Keywords:** trastuzumab, taxanes, tumour targeting, mitochondrial membrane potential, reactive oxygen species (ROS) formation

## Abstract

Taxanes are considered fundamental drugs in the treatment of breast cancer, but despite the similarities, docetaxel (doc) and paclitaxel (ptx) work differently. For this reason, it is interesting to identify mechanisms of antitumor activity of PAMAM dendrimer conjugates that carry docetaxel or paclitaxel and monoclonal antibody trastuzumab, specifically targeted to cells which overexpressed HER-2. For this purpose, the impact on the level of reactive oxygen species, the mitochondrial membrane potential, cell cycle distribution and the activity of caspases-3/7, -8 and -9 of PAMAM-doc-trastuzumab and PAMAM-ptx-trastuzumab conjugates was determined and compared with free docetaxel and paclitaxel toward HER-2-positive (SKBR-3) and negative (MCF-7) human breast cancer cell lines. Moreover, apoptosis and necrosis were studied using flow cytometry and confocal microscopy, respectively. Our studies show the complexity of the potential mechanism of cytotoxic action of PAMAM-drug-trastuzumab conjugates that should be sought as a resultant of oxidative stress, mitochondrial activation of the caspase cascade and the HER-2 receptor blockade.

## 1. Introduction

The history of taxanes begin in the early 1960s with the discovery of the antitumor activity of *Taxus brevifolia* tree bark extract, named paclitaxel (Taxol, ptx) in 1971 [1]. Docetaxel—a semi-synthetic analogue of paclitaxel, which in some cases exhibits better efficacy than paclitaxel—was approved by the FDA for breast cancer treatment in 1996 [2]. In the nineties, paclitaxel and docetaxel were approved for the treatment of other solid tumours, and they are still fundamental in the treatment of advanced and early-stage breast cancer. Discovered in the eighties, the mechanism of action of taxanes demonstrated tubulin stabilisation causing mitotic arrest [3,4]. Taxanes bind to the β-microtubule chain and enhance tubulin polymerisation. Docetaxel and paclitaxel can inhibit mitosis and intracellular transport within cells, leading to apoptotic cell death. Taxanes can also block the BCL-2 gene family and induce p53 gene activation, the consequence of which is mitotic arrest and cell death [5].

As the data from the literature demonstrates, the mechanism of action of taxanes is not limited to microtubule stabilisation, mitotic arrest and apoptotic cell death, and new aspects of these drugs are constantly discovered. Recently, it was shown that taxanes can also affect the androgen receptor (AR) and a significant correlation was found between clinical response to taxane chemotherapy and AR cytoplasmic sequestration in hormone-refractory prostate cancer (HRPC) patients [6].

Taxanes, as well as other chemotherapy drugs, have their limitations, including multidrug resistance (MDR). Since paclitaxel and docetaxel have a high affinity for the ATP-dependent drug efflux pump P-glycoprotein (Pgp) [7], it is considered that Pgp expression by cancer cells can be responsible for resistance to taxanes. Another limitation may be overexpression of class III β-tubulin [8].

These are not the only limitations of taxanes. Despite the clinical progress in the treatment of cancer with taxanes, paclitaxel and docetaxel, their effectiveness is limited by hydrophobicity. Solvent-based delivery vehicles for chemotherapy agents allowing hydrophobic drugs to be administered intravenously are associated with serious toxic side effects [9]. Moreover, both taxanes suffer from the lack of tumour specificity. That is why new solutions are being sought, such as cabazitaxel, which exhibits improved potency against MDR-expressing tumours, but its clinical application is intended for prostate cancer only [10], or abraxane—the albumin-bound paclitaxel nano-droplet formulation—which expanded the clinical application of paclitaxel but is highly, not selectively, cytotoxic [11].

Therefore, drug combination appears to be the most attractive area of pre-clinical research, e.g., abraxane was successfully used with trastuzumab and carboplatin in first-line therapy for advanced HER-2 positive breast cancer [12] and docetaxel with pertuzumab and trastuzumab in first-line treatment for HER2-positive metastatic breast cancer [13]. The question arises what advantages can be achieved using a monoclonal antibody with taxanes? Our previous studies demonstrated the utility of trastuzumab as a targeting agent. Moreover, PAMAM dendrimer conjugates, with trastuzumab and docetaxel or paclitaxel, improved the efficacy of targeted delivery of these anticancer drugs [14]. Therefore, what is so unique in trastuzumab that it has such an impact on increasing the efficiency and selectivity of PAMAM-drug-trastuzumab conjugates?

Trastuzumab is a recombinant humanised monoclonal antibody targeted against the extracellular domain of the HER-2 protein [15]. The HER-2 gene is overexpressed in more than 20% of all primary invasive breast cancers (HER-2-positive breast cancer) [16]. Because HER-2 overexpression is associated with poor disease-free survival, HER-2 gene amplifications are considered to be an independent adverse prognostic factor [17]. Some studies have shown that trastuzumab may increase the efficacy of commonly used chemotherapy as a factor supporting the induction of apoptosis [18]. Furthermore, several possible modes of action of trastuzumab have been proposed in the literature, such as cytotoxicity, inhibition of DNA repair, cell-cycle arrest, suppression of angiogenesis and inhibition of HER-2 extracellular proteolysis [19,20], but the exact mechanism of anticancer activity of trastuzumab alone or in combined therapy with anticancer drugs has not been fully elucidated. Therefore, studies that enable understanding the mechanism of anticancer activity of taxanes and trastuzumab are so important.

Our previous studies showed that application of PAMAM dendrimer conjugation significantly increased cellular uptake of taxanes, enabling passive delivery of paclitaxel or docetaxel, which consequently increased their cytotoxicity [14]. They also showed that trastuzumab can be used in a PAMAM-drug-trastuzumab conjugate carrying paclitaxel (ptx) or docetaxel (doc) to specifically target SKBR-3 HER-2 positive cells. Moreover, PAMAM-drug-trastuzumab conjugates proved increased toxicity toward HER-2-positive human breast cancer cells compared with the free drug or the PAMAM-trastuzumab conjugate.

Since the cytotoxic activity of PAMAM-drug-trastuzumab conjugates and free drugs was previously tested on HER-2-positive (SKBR-3) and negative (MCF-7) human breast cancer cell lines, the same cell lines were used in the present study to investigate the influence of the conjugates and drugs on mitochondrial membrane potential, the intracellular reactive oxygen species generation, caspases activity, cell cycle and ability to induce apoptosis or necrosis.

The results presented in this article are the next step to a better understanding of the mechanism responsible for the enhanced therapeutic effect of taxanes and selectivity of their conjugates with trastuzumab in comparison with the free drugs. The analysed drugs and their conjugates showed a different mechanism of action. The PAMAM-doc-trastuzumab conjugate generated more reactive oxygen species than the free docetaxel, while conversely, the PAMAM-ptx-trastuzumab conjugate had a lesser influence on the intracellular ROS level than free paclitaxel. In the case of mitochondrial membrane potential, the PAMAM-ptx-trastuzumab conjugate acted similar to free taxanes, triggering at first an increased, and then a decreased, mitochondrial membrane potential, in contrast to the PAMAM-doc-trastuzumab conjugate, which evoked immediate mitochondrial depolarisation. Interestingly, only in the case of SKBR-3 cells, both PAMAM-drug-trastuzumab conjugates maintained the mechanism of action of free drugs and activated the caspase cascade, which was reflected in higher apoptosis induction. The most important finding was that all components of the conjugates, only when acting together, can achieve a higher efficacy and selective toxicity.

## 2. Materials and Methods

### 2.1. Materials

Solvents for the synthesis and purification were purchased from Sigma-Aldrich (Poznan, Poland). All cell culture reagents were purchased from Gibco^®^ (Life Technologies Polska Sp. z o. o., Warsaw, Poland). Flasks and multiwell transparent and black plates for in vitro studies were obtained from Nunc (Life Technologies Polska Sp. z o. o., Warsaw, Poland). PAMAM G4-NH_2_ dendrimer, docetaxel, paclitaxel, phosphate buffered saline (PBS), fetal bovine serum (FBS), propidium iodide (PI) and ribonuclease A deoxyribonuclease-free were purchased from Sigma-Aldrich (Poznan, Poland). Trypan blue was purchased from Molecular Probes ((Thermo Scientific™, Warsaw, Poland). Annexin V and 2′,7′-dichlorodihydrofluorescein (H2DCF-DA) were purchased from BD Biosciences (Warsaw, Poland). Caspase-Glo^®^ 3/7 Assay, Caspase-Glo^®^ 8 Assay and Caspase-Glo^®^ 9 Assay systems were purchased from Promega Corporation (Mannheim, Germany). Trastuzumab (Herceptin) was obtained from Roche Poland (Poznan, Poland). Human breast adenocarcinoma′s cell lines, including HER-2 positive (SKBR-3 ATCC no. HTB-30) and HER-2 negative (MCF-7 ATCC no. HTB-22) were purchased from ATCC (LGC Standards Sp. z o. o., Lomianki, Poland).

### 2.2. Synthesis of Conjugates

The linking of the taxanes to the PAMAM dendrimer was done using a two steps covalent method (patent pending P.420273) while the synthesis of PAMAM-doc-trastuzumab and PAMAM-ptx-trastuzumab conjugates was performed according to the methods (patent pending P.420274) described earlier [14]. The stoichiometric ratio of PAMAM-drug conjugate was 1:1 and PAMAM-drug-trastuzumab conjugate was 1:1:1, respectively.

### 2.3. Cell Culture

MCF-7 (HER-2 negative human breast adenocarcinoma cell line) was cultured in DMEM medium enriched with 10% (*v*/*v*) foetal bovine serum (FBS) and GlutaMAX. SKBR-3 (HER-2 positive human breast adenocarcinoma cell line) was cultured in McCoy′s 5A medium also supplemented with GlutaMAX and 10% (*v*/*v*) FBS. Cells were cultured in T-75 culture flasks in the atmosphere containing 5.0% CO_2_ at 37 °C and subcultured every 2–3 days. Cells were used in experiments after obtaining 80–90% confluence. The number of viable cells was determined by the trypan blue exclusion assay using a Invitrogen Countess Automated Cell Counter (Life Technologies Polska Sp. z o. o., Warsaw, Poland). Cells were seeded in flat bottom 96-well transparent plates at a density of 2.0 × 10^4^ cells/well in 100 μL of an appropriate medium or in flat bottom 12-well transparent plates at a density of 2.0 × 10^5^ cells/well in 1 mL of an appropriate medium. After seeding, plates were incubated for 24 h in a humidified atmosphere containing 5.0% CO_2_ at 37 °C.

### 2.4. Measurement of Reactive Oxygen Species (ROS)

Changes in the level of reactive oxygen species were checked using a fluorescent probe 2′,7′-dichlorodihydrofluorescein. The biological mechanism of the probe activity is the elimination of acetate groups by intracellular esterases, followed by oxidation of the compound to dichlorofluorescein (DCF) [21]. Cells were seeded on 96-well black plates at a density of 2.0 × 10^4^ cells/well in 100 μL of an appropriate medium. After treatment, cells were stained with 2 μM H_2_DCFDA for 15 min in growing conditions. Subsequently the dye solution was removed and cells were washed with PBS. Fluorescence (λ_ex_ = 485 nm, λ_em_ = 530 nm) was measured using the Synergy™ HTX Multi-Mode Microplate Reader (BioTek, Winooski, VT, USA).

### 2.5. Assessment of Mitochondrial Membrane Potential (ΔΨm)

Mitochondrial membrane potential was measured using a JC-1 fluorescent lipophilic cationic dye, which at higher concentrations forms J-aggregates accumulating in mitochondria and exhibits red fluorescence (λ_ex_ = 530 nm, λ_em_ = 590 nm). When the mitochondrial membrane is depolarized, the dye does not aggregate but exists in the form of monomers, which emit green fluorescence (λ_ex_ = 485 nm, λ_em_ = 540). The loss of ΔΨ_m_ can be indicated by a decrease in the red to green fluorescence intensity ratio [22]. Cells were seeded on 96-well black plates at a density of 2.0 × 10^4^ cells/well in 100 μL of an appropriate medium. After treatment, 50 μL of 5 μM JC-1 was added to each well and incubated for 30 min in growing conditions. The dye was removed, cells were washed with PBS and then 50 μL of PBS was added to each well. Measurements were performed using the Synergy™ HTX Multi-Mode Microplate Reader.

### 2.6. Measurement of Caspases Activity

Estimates of the activity of caspases-3/7, -8 and -9 were performed using assay kits (Caspase-Glo^®^ 3/7 Assay, Caspase-Glo^®^ 8 Assay and Caspase-Glo^®^ 9 Assay systems Promega Corporation) according to the producer recommendations. 

Cells were seeded on 96-well black plates at a density of 2.0 × 10^4^ cells/well in 100 μL of an appropriate medium. After treatment cells were centrifuged and 50 µL of supernatant was transferred to new 96-well black plate. Then 50 μL of the reaction mixture was added to each well and incubated for 20/40/60 min in growing conditions. The chemiluminescence was read using the Synergy^™^ HTX Multi-Mode Microplate Reader.

### 2.7. Detection of Apoptotic and Necrotic Cells

Annexin V and propidium iodide (PI) staining was performed to detect apoptotic and necrotic cells. Annexin V is a protein that specifically binds to phosphatidylserine, which is translocated from the inner layer of the plasma membrane to the outer layer during early apoptotic cells. The cell membrane of apoptotic cells stained with Annexin V conjugated with fluorescein isothiocyanate (FITC) is not permeable to the red fluorescent dye propidium iodide, which is able to penetrate the interior of necrotic cells. For this reason, the method is suitable to distinguishing between intact, apoptotic and necrotic cell populations. A low level of green fluorescence is characteristic for viable cells. Apoptotic cells show an increased level of green fluorescence, while necrotic cells exhibit both red and green fluorescence [23]. Cells were seeded in 24-well transparent plates. After treatment, cells were trypsinised, and then washed with PBS and suspended in 500 µL of binding buffer (delivered from the producer). The mixture consisting of 5 µL of Annexin V conjugated with FITC and 5 µL of propidium iodide was added to cell suspension. Samples were incubated at room temperature for 20 min in the dark. Measurement of fluorescence intensity was performed by a Becton Dickinson LSR II flow cytometer. The control apoptosis was induced by camptothecin (80 µM) and necrosis was induced by pentachlorophenol (0.6 µM) (data not shown). The data were recorded for a total of 10,000 events per sample.

### 2.8. Confocal Microscopy

Confocal microscopy images were obtained using confocal inverted microscope SP-8, Leica equipped with 405 nm laser (Labsoft Sp. z o.o., Warsaw, Poland). Cells at the density of 1 × 10^4^ cells/well (SKBR-3) and 0.75 × 10^4^ cells/well (MCF-7) were seeded on 96-well glass-bottom plates and incubated with 1 µM docetaxel or paclitaxel or PAMAM-doc-trastuzumab or PAMAM-ptx-trastuzumab conjugate for 24 h in 37 °C humidified atmosphere containing 5.0% CO_2_. After treatment, cells were washed with PBS, and then suspended in 100 µL of binding buffer (delivered from the producer). The mixture consisting of 1 µL of Annexin V fluorescein isothiocyanate and 1 µL of propidium iodide was added to cell suspension. Samples were incubated at room temperature for 20 min in the dark. Thereafter, cells were cooled on ice and washed once with cold phosphate buffered saline. Cells were imaged to visualize fluorescence of FITC labelled Annexin V in the green channel (excitation 488 nm, emission 520 nm) and propidium iodide in the red channel (excitation 535 nm, emission 617 nm).

### 2.9. Cell Cycle Studies 

Cell cycle distribution was analysed by flow cytometry (LSRII; Becton Dickinson Biosciences, San Jose, CA, USA) after propidium iodide staining according to Chang et al. [24]. Cells were seeded in 24-well plates. After treatment cells were trypsinised, collected and fixed in ice-cold 96% ethanol for 24 h. Then, cells were washed with PBS and incubated for 30 min at 4 °C in 500 μL of staining solution containing 10 mM Tris-HCl (pH = 7.5), 5 mM magnesium chloride, 10 μg/mL propidium iodide and 10 μg/mL ribonuclease A. After this time samples were analysed by a Becton Dickinson LSRII flow cytometer. The data were recorded for a total of 10,000 events per sample.

### 2.10. Statistical Analysis

Data was expressed as mean ± SD. Analysis of variance (ANOVA) with the Tukey post hoc test was used for results comparison. All statistics were calculated using the Statistica software (StatSoft, Tulsa, OK, USA), and *p* < 0.05 was considered significant.

## 3. Results and Discussion

Our previous studies showed that PAMAM-drug-trastuzumab conjugates possess increased toxicity toward HER-2-positive human breast cancer cells compared with the free drug or the PAMAM-trastuzumab conjugate. The IC_50_ values in SKBR-3 cells were equal to 0.004 µM for the PAMAM-doc-trastuzumab conjugate and 0.002 µM for the PAMAM-ptx-trastuzumab conjugate after a 48 h incubation compared with the 2 µM concentration of free docetaxel and 0.49 µM of free paclitaxel, respectively. However, the most remarkable observation was the selectivity between cell lines, especially for the PAMAM-doc-trastuzumab conjugate (48.85 µM for MCF-7 HER-2-negative and 0.004 µM for SKBR-3 HER-2-positive cell lines) [14]. The IC_50_ value for SKBR-3 cells confirmed also the increase in selectivity and therapeutic effect compared not only to free drugs but also to the PAMAM-trastuzumab conjugate (0.41 ± 0.06 µmol/L) [25]. It is important to mention that toxicity of the cationic amino-terminated PAMAM dendrimer generation 4 had no impact on the overall PAMAM-drug-trastuzumab conjugates cytotoxicity in the 0.002–0.004 µM concentration range, since our previous studies proved that for this dendrimer the IC_50_ values obtained for sensitive Chinese hamster ovary (CHO) and resistant human ovarian carcinoma (SKOV3) cell lines were 5.56 and 46.49 µM, respectively [26]. High toxicity is usually correlated with a higher production of ROS, and finally, induction of cell death. The interaction of most anticancer drugs with the mitochondria led to ROS production. Moreover, the intracellular ROS play an important role in apoptosis through affecting several signalling pathways [27,28]. Since oxidative stress has an influence on mitochondria, the cellular redox homeostasis is a crucial factor in the modulation of apoptosis [26]. For this reason, it is very important to check whether the PAMAM-drug-trastuzumab conjugates can initiate oxidative resulting from cell overproduction of ROS. 

**Figure 1:**
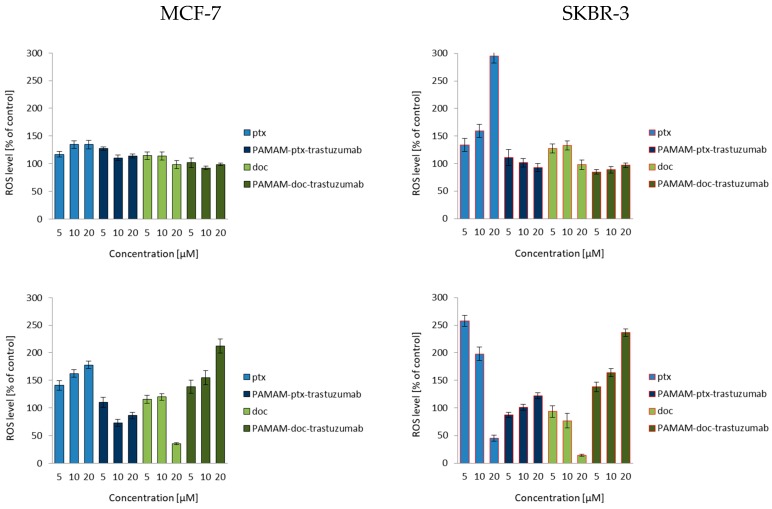
Influence of the PAMAM-ptx-trastuzumab conjugate, PAMAM-doc-trastuzumab conjugate, paclitaxel (ptx) and docetaxel (doc) at a concentration range of 5–20 μM on the ROS generation in MCF-7 cells (**left panel**) and SKBR-3 cells (**right panel**) after 3 and 24 h incubation periods. The results are presented as the mean ± standard deviation of three experiments.

To fully understand the mechanisms of HER-2-negative and HER-2-positive cell death, ROS production was estimated by the formation of a highly fluorescent dichlorofluorescein (DCF), the compound formed after the oxidation of non-fluorescent H2DCF-DA by cytosolic esterases [21]. As shown in Figure 1, after a 3 h incubation, only free drugs enhanced the production of ROS, especially the highest 20 µM paclitaxel concentration in the SKBR-3 cell line. After a 24 h incubation, some of the cells treated with free drugs were already dead, while PAMAM-drug-trastuzumab conjugates were just starting to act. Our measurements of ROS formation brought interesting findings. Considering the SKBR-3 cell line (Figure 1, right panel), paclitaxel induced a higher level of ROS than the more toxic PAMAM-ptx-trastuzumab conjugate. Some reports have indicated that taxanes only provoke a low level of oxidative stress and they generate some ROS, but the apoptosis they trigger in cancer cells is mainly by the release of cytochrome c from mitochondria [29]. However, one must bear in mind that paclitaxel is not soluble in water, but in DMSO, and therefore, the observed effect might be enhanced by the solvent. In the case of the conjugates, PAMAM dendrimers may affect the ROS production because, on the one hand, they are the substitute for the solvent, but on the other hand, they have an impact on mitochondria. Mukherjee et al. showed that the interaction of amino-terminated PAMAM dendrimers with the mitochondria led to ROS production [30]. Mohan et al. demonstrated that trastuzumab significantly enhanced the generation of intracellular ROS, peroxides and free radicals in cardiomyocytes [31]. Thus, one would expect that the observed effect of the conjugate should be the resultant of the action of these three components: The anticancer drug, the PAMAM dendrimer carrier and the monoclonal antibody trastuzumab. However, the PAMAM-ptx-trastuzumab conjugate induced only a low level of ROS in comparison to free paclitaxel. Interestingly, the PAMAM-doc-trastuzumab conjugate generated more ROS than the PAMAM-ptx-trastuzumab conjugate, but after a 24 h incubation. This may be the first step in indicating a difference in the mechanism of action of these conjugates.

Mitochondria activation most often leads to changes in the mitochondrial membrane potential, permeability transition, increased production of intracellular ROS and the generation of apoptotic proteins in the cell. The change of mitochondrial membrane potential can cause cell death. Zorov et al. described mechanisms assuming that an increase in ROS generation reaching a threshold level which caused the opening of the channels in mitochondrial membrane, resulted in the collapse of its potential and increased ROS production [32]. Mitochondrial depolarisation—a decrease in the mitochondrial membrane potential—occurs in the early stages of apoptosis and is preceded by mitochondrial hyperpolarisation, relying on the locking of the voltage dependent anion channel (VDAC) with the Bax peptide [33] or by generating a proton gradient across the inner membrane [34]. 

**Figure 2:**
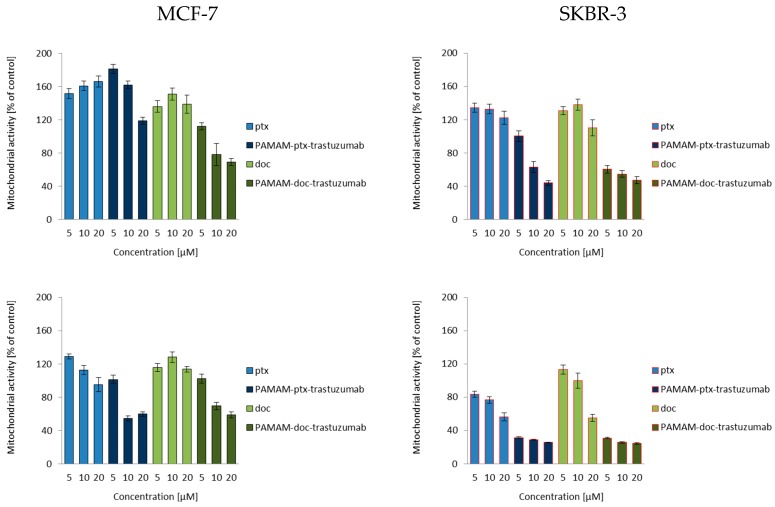
Influence of the PAMAM-ptx-trastuzumab conjugate, PAMAM-doc-trastuzumab conjugate, paclitaxel (ptx) and docetaxel (doc) on the mitochondrial membrane potential in MCF-7 cells (**left panel**) and SKBR-3 cells (**right panel**) after 3 and 24 h incubation. The results are presented as the mean ± standard deviation of three experiments.

According to current knowledge on the action of taxanes, after a 3 h incubation, both docetaxel and paclitaxel increased the mitochondrial membrane potential only to decrease it after a 24 h incubation (Figure 2). Many in vitro studies have showed that paclitaxel and docetaxel are able to alter the mitochondrial structure, and their function can evoke mitochondrial depolarisation due to the opening of the mitochondrial permeability transition pore (mPTP) and release of Ca^2+^ from mitochondria [35,36,37]. An amino-terminated PAMAM G4 dendrimer can also decrease the mitochondrial membrane potential in a concentration-dependent manner, where the higher the toxicity of the dendrimer the higher the depolarisation of the mitochondrial membrane [38]. Since trastuzumab can trigger cellular oxidative stress and induce apoptosis through the depolarisation of mitochondria, the action of all mentioned compounds together should also lead to mitochondrial defects, causing the opening of the mPTP and the activation of cell death pathways. Figure 2 summarises the results of the experiment devoted to the analysis of the ability of free paclitaxel and docetaxel, as well as PAMAM-drug-trastuzumab conjugates, to induce changes in mitochondrial membrane potential. As it can be observed in the case of the MCF-7 HER-2-negative cell line, there were significant differences between the PAMAM-drug-trastuzumab conjugates. The PAMAM-ptx-trastuzumab conjugate acted similar to free drugs, initially triggering an increase, followed by a decrease of the mitochondrial membrane potential, in contrast to the PAMAM-doc-trastuzumab conjugate, which evoked an immediate mitochondrial depolarisation. Interestingly, in the case of the SKBR-3 HER-2-positive cell line, both conjugates evoked very high mitochondrial depolarisation, especially after a 24 h incubation.

Analysing changes in mitochondrial potential, we noticed differences in the effects of free drugs and conjugates depending on the time of drug-treatment and the cell line. However, very large changes caused by conjugates compared to free drugs in the SKBR-3 HER-2-positive cell line confirmed our previous results of selective cytotoxicity and may suggest an even greater effect of the tested compounds on the activation of caspases and apoptosis.

One of the factors released from the mitochondria during depolarisation was cytochrome c (Apaf-2). In the cytoplasm, Apaf-2 combines with the protein factor Apaf-1 and procaspase-9 to form a structure called apoptosome. The main task of the apoptosome is to activate executive caspases [39]. Activation of procaspase-9 on the apoptosome is a crucial step in the intrinsic cell death pathway. Executive caspases are also essential for apoptosis-associated chromatin condensation, DNA fragmentation and nuclear collapse [40]. Second, a major cellular pathway of drug-induced apoptosis is the CD95 death receptor pathway, initiated by ligation of the death receptor by its ligand CD95L [41].

The release of cytochrome c and ligation of proapoptotic signals of CD95 activate initiator caspases, caspases-8 and -9, respectively. Then the initiator caspases activate the effector caspases, caspases-3, -6 and -7, which lead to the induction of apoptosis. 

**Figure 3:**
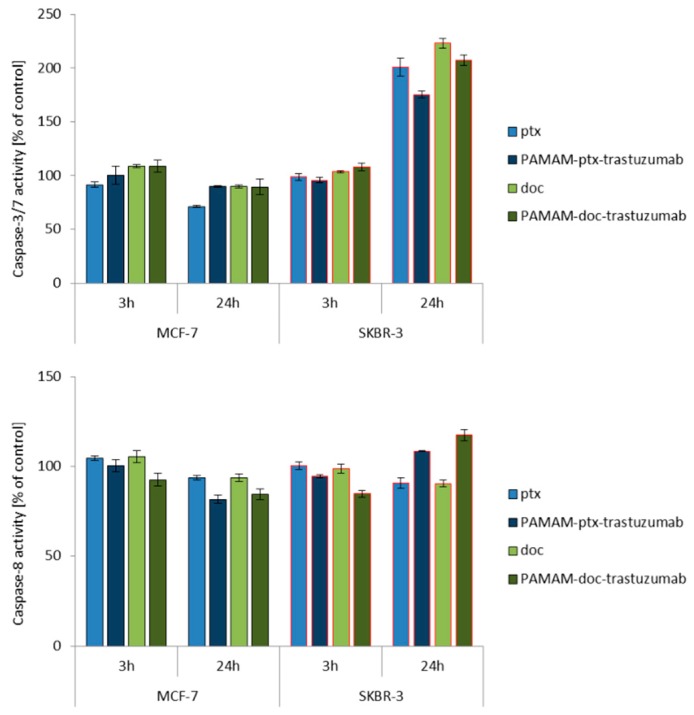

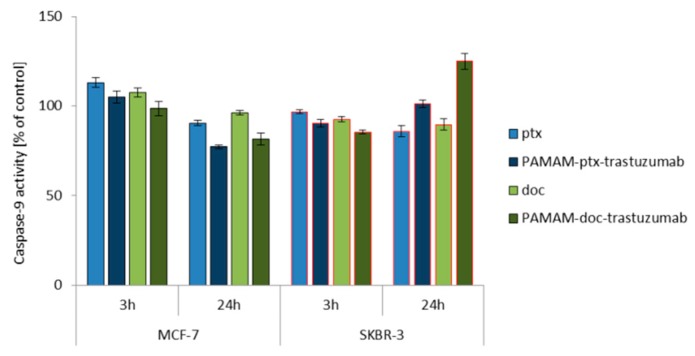
Influence of the PAMAM-ptx-trastuzumab conjugate, PAMAM-doc-trastuzumab conjugate, paclitaxel (ptx) and docetaxel (doc) on the caspases 3/7, 8 and 9 activation in MCF-7 and SKBR-3 cells after a 3 and 24 h incubation period. The results are presented as the mean ± standard deviation of three experiments.

Since caspase activation occurs almost immediately after ROS generation (oxidative damage → caspases-8) or changes in mitochondrial membrane potential (mitochondria damage → caspases-9), the activity of caspase-8, -9 and -3/7 was studied at the same time points (3 and 24 h) for a 1 μM dose of free paclitaxel and docetaxel, as well as the PAMAM-drug-trastuzumab conjugates. For all analysed compounds, a biphasic caspase activity was observed (Figure 3), similar to that caused by polyamidoamine dendrimer nanoparticles [38]. For free paclitaxel and docetaxel, an initial increase of caspases levels was observed after a 3 h incubation, while a decrease was observed after a 24 h incubation, except for caspase-3/7, which reached a maximum level in the SKBR-3 line after a 24 h incubation period with the drugs. A similar behaviour was observed for the PAMAM-drug-trastuzumab conjugates. In the MCF-7 cell line, both conjugates resulted in a decrease in the activity of all caspases, after reaching the initial maximum. In contrast, in the SKBR-3 cell line, the activity of all caspases increased to reach a maximum after a 24 h incubation period, especially caspase-3/7.

This is a very important result, because it was shown in the case of SKBR-3 cells that both PAMAM-drug-conjugates maintain the mechanism of action of free drugs and activate initiator caspases-8 and -9, which participate in the activation of the caspase cascade—and of particular importance, caspase-3—which is necessary for cleavage of the majority of substrates examined, as well as DNA fragmentation and nuclear collapse resulting in apoptotic cell death [42]. Our results are in line with other studies showing the involvement of caspase activation in taxane-induced apoptosis of cancer cells derived from the breast cancer paclitaxel-sensitive MDA-MB-435 and paclitaxel-resistant NCI/ADR-RES cell lines [43]; the human pulmonary adenocarcinoma A549 cell line [44]; the ovarian cancer paclitaxel-resistant HER-2/neu-overexpressing SKOV3.ipl cell line [45]; and the prostate cancer DU145 cell line [46]. However, here arises the question: Why do the free drugs and conjugates, which in the MCF-7 cell line caused high mitochondrial membrane hyperpolarisation, not activate the caspase cascade in this cell line as strongly as they did in the SKBR-3 cell line? The precise reason for this inconsistency is not known, but we presume that the inconsistency might be due to cellular differences in tissue origin, status of differentiation or cell cycle checkpoint/regulatory proteins such as p53 [47]. Our hypothesis can be confirmed by other studies showing that, especially paclitaxel-induced apoptosis, is not always related to caspase activity, just like in the case of the lung cancer cell line NCI-H460, ovarian cancer cell line SKOV3 or the previously mentioned breast cancer MCF-7 cell line [48,49]. 

**Figure 4:**
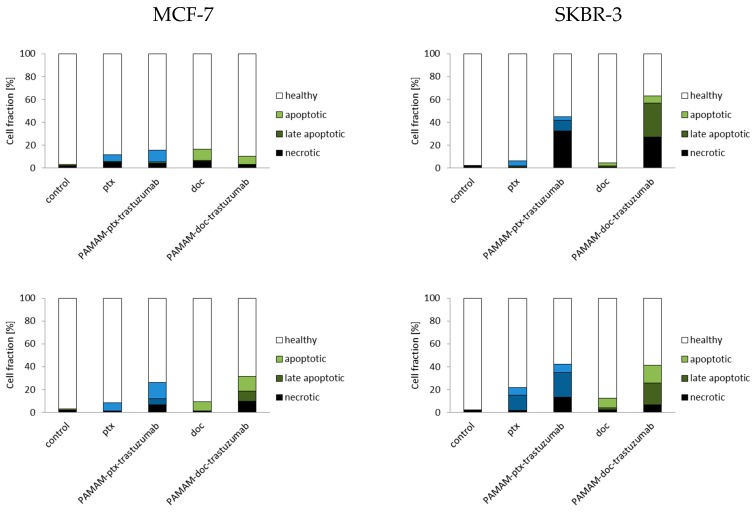
Fractions of apoptotic and necrotic cells after 3 and 24 h incubation periods with the PAMAM-ptx-trastuzumab conjugate, PAMAM-doc-trastuzumab conjugate, paclitaxel (ptx) and docetaxel (doc) at a 1 µM concentration in MCF-7 cells (**left panel**) and SKBR-3 cells (**right panel**). The results are presented as the mean ± standard deviation of three experiments.

Since the results from this study indicate that PAMAM-drug-trastuzumab-induced apoptosis in SKBR-3 HER-2-positive cell line is associated with the caspase-dependent pathway, we decided to check fractions of apoptotic and necrotic cells after 3 and 24 h incubation periods with the PAMAM-ptx-trastuzumab conjugate and the PAMAM-doc-trastuzumab conjugate, as well as paclitaxel and docetaxel in both cell lines (Figure 4). Apoptosis is a form of programmed cell death that is characterised by condensation of the nuclear chromatin, cell shrinkage, DNA fragmentation, exposure of apoptotic bodies and changes in the symmetry of phosphatidylserine (PS). In contrast to apoptosis, necrosis is demonstrated by a loss of membrane integrity, shut down of metabolism and the release of cytoplasmic components [26]. The cell membrane of apoptotic cells stained with Annexin V conjugated with fluorescein isothiocyanate (FITC) is impermeable compared to the red fluorescent dye propidium iodide (PI), which can penetrate the interior of necrotic cells. As it can be observed in Figure 4, in the case of MCF-7 HER-2-negative cell line and the SKBR-3 HER-2-positive cell line, after 3 h, the conjugates were more effective than free drugs, resulting in a higher percent of early and late apoptotic cells (12.1% of cells for PAMAM-ptx-trastuzumab and 7.3% of cells for PAMAM-doc-trastuzumab in the MCF-7 cell line; 12.3% of cells for PAMAM-ptx-trastuzumab and 35.9% of cells for PAMAM-doc-trastuzumab in SKBR cell line, respectively), as compared to free drugs (10.9% of cells for paclitaxel and 12.0% of cells for docetaxel in MCF-7 cell line; 5.5% cells for paclitaxel and 3.7% cells for docetaxel in SKBR cell line, respectively). 

Analysing the results after 24 h of incubation, it should be considered that some of the dead cells were too distorted to be measured by the flow cytometry. Therefore, the only information that is important is the fact that the fraction of early and late apoptotic cells after 24 h increased for both conjugates, especially for the PAMAM-ptx-trastuzumab conjugate in the SKBR cell line (15.3% of cells for the PAMAM-ptx-trastuzumab and 15.8% of cells for the PAMAM-doc-trastuzumab in the MCF-7 cell line; 28.5% of cells for the PAMAM-ptx-trastuzumab and 36.4% of cells for the PAMAM-doc-trastuzumab in the SKBR cell line, respectively). The results indicate that in the case of the PAMAM-doc-trastuzumab conjugate, the major mechanism of cell death is apoptosis. Kulhari et al.′s research also confirms that dendrimer conjugated with trastuzumab and docetaxel (TZ-Dend-DTX) is able to induce higher level of apoptosis than free docetaxel (DTX) [50]. In the case of the PAMAM-ptx-trastuzumab conjugate, the leading mechanism is initially necrosis, and after prolonged incubation, apoptosis. This is in line with our previous observations that in the case of a paclitaxel conjugate, other mechanisms may also be involved in the cell death process. Annexin V studies confirm that trastuzumab targeting is crucial for the enhanced cytotoxic activity of PAMAM-drug-trastuzumab conjugates [14].

**Figure 5:**
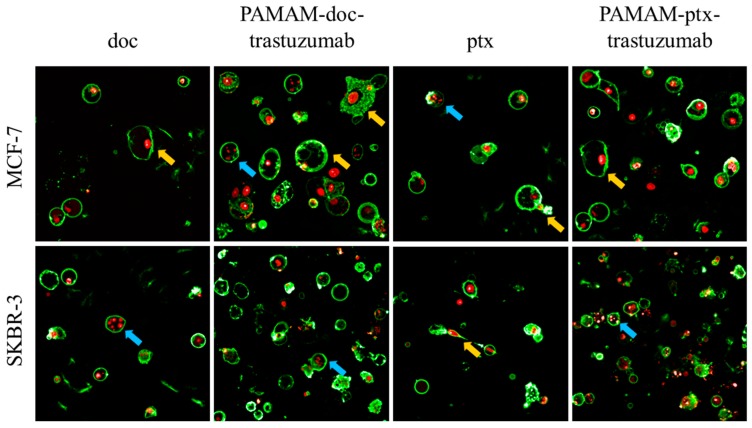
Representative confocal images of MCF-7 and SKBR-3 cells after a 24 h incubation period with the analysed compounds. The cells were stained with Annexin V and propidium iodide (PI). After drug treatment, a strong green fluorescence (derived from Annexin-V FITC) in the cells with phosphatidylserine translocated from the inner layer of the plasma membrane to the outer layer was noticed, which is characteristic for symptoms of programmed cell death. Cancer cells treated with investigated drugs present abnormal morphology: Chromatin condensation and fragmentation (marked by the blue arrows 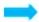), giant cells and elongated cells with protrusions from the plasma membrane and cytoplasmic bridges between cells (marked by the yellow arrows 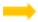). For interpretation of the references to colour in this figure legend, the reader is referred to the web version of this article.

The results obtained from the analysis of fractions of apoptotic and necrotic cells were confirmed with confocal images (Figure 5). Applying confocal microscopy, it was shown that after a 24 h incubation period, both PAMAM-drug-trastuzumab conjugates caused numerous changes typical for apoptosis, but also for necrosis: Alteration in the structure, size and shape of the cell’s nucleus, profound chromatin condensation, cell shrinkage and nuclear fragmentation, formation of apoptotic bodies, impairment of the plasma membrane and cell disintegration. 

**Figure 6:**
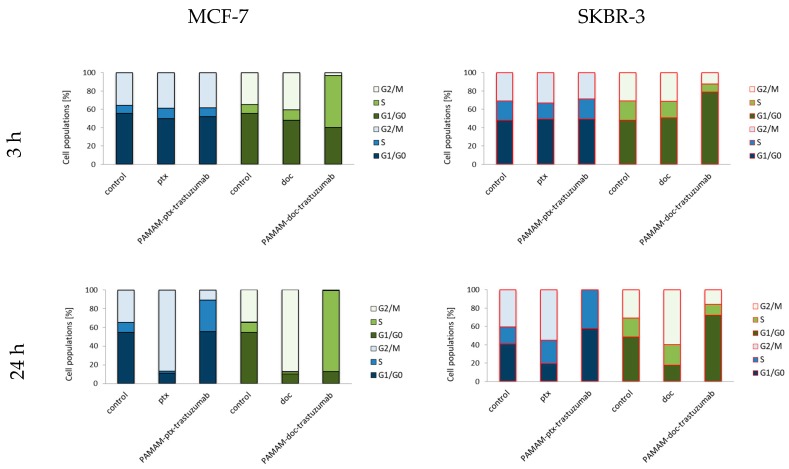
Cell cycle analysis for control (untreated cells) and cells treated with 1 µM of the PAMAM-ptx-trastuzumab conjugate, PAMAM-doc-trastuzumab conjugate, paclitaxel (ptx) and docetaxel (doc) in MCF-7 cells (**left panel**) and SKBR-3 cells (**right panel**) after 3 and 24 h incubation periods. The results are presented as the mean of three experiments.

In addition to the generation of oxidative stress and the induction of apoptosis, another aspect of the action of anticancer drugs is the impact on the cell cycle. The cell cycle consists of several phases: G1–preparation for DNA synthesis; S–phase of DNA synthesis; G2–preparation for mitosis; and M–mitosis during which the cell divides into two similar cells. In these phases, there are also the checkpoints that ensure the integrity of the genome. The G1 and G2 checkpoints arrest the cell cycle when DNA damage is detected; in the S phase checkpoint, the cycle arrest is the result of a problem with DNA replication; and the M phase checkpoint is responsible for cell arrest if a problem with the mitotic spindle assembly occurs [51]. Taxanes interfere with the mitotic process and therefore they act mostly during the M phase, while trastuzumab influence the cell cycle causing cell arrest in the G1/S checkpoint [51,52]. Figure 6 summarises the results of the cell cycle analysis for control (untreated) cells and cells treated with free paclitaxel and docetaxel, as well as PAMAM-drug-trastuzumab conjugates at a 1 µM concentration after a 3 and 24 h incubation period. In the case of the MCF-7 HER-2-negative cell line, after a 3 h incubation period, docetaxel only caused a slight cycle arrest in the G2/M phase, while the PAMAM-doc-trastuzumab conjugate blocked as much as 56.6% of cells in the S phase. Interestingly, paclitaxel and its conjugate had no influence on the cell cycle. However, after a 24 h incubation, docetaxel and paclitaxel caused cell cycle arrest in the G2/M phase (87.4% and 86.7%, respectively), and both conjugates increased cell accumulation in the S phase (PAMAM-doc-trastuzumab to 86.8% and PAMAM-ptx-trastuzumab to 33.5%). In the case of the SKBR-3 HER-2-positive cell line after a 3 h incubation period, both free taxanes and the PAMAM-ptx-trastuzumab conjugate did not affect the cell cycle, only the PAMAM-doc-trastuzumab conjugate induced significant cell accumulation in the G1 phase. After a 24 h incubation, paclitaxel and docetaxel increased cell accumulation in the S phase (slight) and in G2/M phase (significant), while the PAMAM-ptx-trastuzumab conjugate caused cell cycle arrest in the G1 and S phases (57.6% and 42.3%, respectively) and the PAMAM-doc-trastuzumab conjugate only caused cell cycle arrest in the G1 phase (72.5%). The results obtained for PAMAM-drug-trastuzumab conjugates are in good agreement with other data where trastuzumab has been found to interfere with HER-2 receptor signalling resulting in cell arrest at the G1/S checkpoint [53,54]. What is important in our study is that we showed that despite the reduction in the HER-2 receptors postulated in SKBR-3 cells, to the level that requires further trastuzumab efficacy [55], our conjugates enabled the trastuzumab to remain effective, and thanks to the combined action with taxanes, our PAMAM-drug-trastuzumab conjugates showed dose-dependent cytotoxicity and differences in molecular mechanisms of their antitumor activity.

The result, which was surprising to us was the accumulation of MCF-7 cells in the S phase caused by the PAMAM-doc-trastuzumab conjugate and in the G1 and S phases caused by the PAMAM-ptx-trastuzumab conjugate, particularly after a 24 h incubation period. Even if docetaxel can also affect centrosome organisation in the S phase, resulting in incomplete mitosis, then paclitaxel should directly affect the mitotic spindle causing cell arrest in the G2/M phase [56]. A different pharmacological mechanism of taxanes regulation also does not explain the stronger activity of the conjugates compared to free drugs. Therefore, in our opinion, the explanation of this mechanism should be sought in the combined action of taxanes with other components of the conjugates. According to Collins et al.’s research, trastuzumab can induce a significant antibody-dependent cell-mediated cytotoxicity response not only in the HER-2-positive HCC1954 and SKBR-3 cell lines, but also in five different HER-2-negative CAL-51, CAMA-1, MCF-7, T47D and EFM19 cell lines, all with the non-amplified HER-2 receptor, detectable only by western blot. Their results showed that despite the low level of HER-2 receptors in normal breast tissue, trastuzumab can bind to HER-2 non-amplified cells and induce a tumour-specific trastuzumab cytotoxicity response [57]. Moreover, some in vitro studies and even clinical trials reported that trastuzumab achieved synergistic interactions with paclitaxel or docetaxel in the MCF-7 HER-2-negative cell line, while only additive interactions in the SKBR-3 HER-2-positive cell line [58,59].

**Scheme 1:**
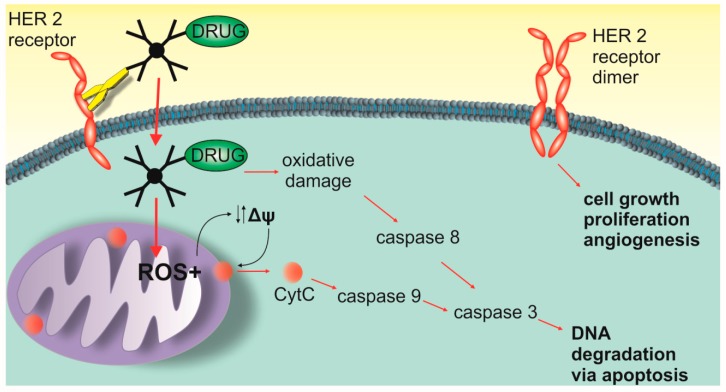
Three main modes of action of the PAMAM-ptx-trastuzumab and PAMAM-doc-trastuzumab conjugate.

Our studies show the complexity of the potential mechanism of cytotoxic action of PAMAM-drug-trastuzumab conjugates. Therefore, we propose three main modes of action for them (Scheme 1). The first mechanism is oxidative stress that can lead to the generation of free radicals, the second is the mitochondrial activation of the caspases cascade and the third mechanism is associated with the blocking of the HER-2 receptor. The combination of these three modes of action may explain the complex mechanism of the selective action of PAMAM-drug-trastuzumab conjugates.

## 4. Conclusions

Taxanes are considered fundamental drugs for breast cancer treatment, but despite their similarities, docetaxel and paclitaxel work differently. Both drugs bind to tubulin, but docetaxel binds stronger than paclitaxel; both drugs promote stabilisation of microtubules but in a different pattern; and both cause G2/M cell cycle arrest leading to cell death, but docetaxel has a more potent antitumor activity. The analysed PAMAM-drug-trastuzumab conjugates showed a different mechanism of action, but the most important finding is that all components of the conjugates, only when acting together, were able to achieve a higher efficacy and selective toxicity. However, bearing in mind that a different response to the drug may occur in cancer patients treated in the same way, and that pharmacodynamics of drugs depend on the complex interactions between genetic and epigenetic factors, our research should be continued in vivo.
